# Exome Sequencing of a Pedigree Reveals S339L Mutation in the *TLN2* Gene as a Cause of Fifth Finger Camptodactyly

**DOI:** 10.1371/journal.pone.0155180

**Published:** 2016-05-25

**Authors:** Hao Deng, Sheng Deng, Hongbo Xu, Han-Xiang Deng, Yulan Chen, Lamei Yuan, Xiong Deng, Shengbo Yang, Liping Guan, Jianguo Zhang, Hong Yuan, Yi Guo

**Affiliations:** 1 Center for Experimental Medicine and Department of Neurology, the Third Xiangya Hospital, Central South University, Changsha, China; 2 Department of Pharmacy, Xiangya Hospital, Central South University, Changsha, China; 3 Division of Neuromuscular Medicine, Davee Department of Neurology and Clinical Neurosciences, Northwestern University Feinberg School of Medicine, Chicago, United States of America; 4 Beijing Genomics Institute-Shenzhen, Shenzhen, China; 5 Department of Dermatology, the Third Xiangya Hospital, Central South University, Changsha, China; 6 Information Security and Big Data Research Institute, Central South University, Changsha, China; German Cancer Research Center (DKFZ), GERMANY

## Abstract

Camptodactyly is a digit deformity characterized by permanent flexion contracture of one or both fifth fingers at the proximal interphalangeal joints. Though over 60 distinct types of syndromic camptodactyly have been described, only one disease locus (3q11.2-q13.12) for nonsyndromic camptodactyly has been identified. To identify the genetic defect for camptodactyly in a four-generation Chinese Han family, exome and Sanger sequencings were conducted and a missense variant, c.1016C>T (p.S339L), in the talin 2 gene (*TLN2*) was identified. The variant co-segregated with disease in the family and was not observed in 12 unaffected family members or 1,000 normal controls, suggesting that p.S339L is a pathogenic mutation. Two asymptomatic carriers in the family indicated incomplete penetrance or more complicated compensated mechanism. Most of p.S339L carriers also have relatively benign cardiac phenotypes. Expression of wild and mutant TLN2 in HEK293 cells suggested the predominant localization in cytoplasm. Our data suggest a potential molecular link between *TLN2* and camptodactyly pathogenesis.

## Introduction

Camptodactyly (MIM 114200) is a digit deformity characterized by permanent flexion contracture of one or both fifth fingers at the proximal interphalangeal (PIP) joints. Additional fingers might be affected, but the little finger is always involved [1]. It is a congenital hand disorder and the prevalence of camptodactyly is between 0.1% and 0.2% [2,3]. Clinically, camptodactyly is usually divided into three types. Type I is the most common, and camptodactyly is limited to the fifth finger. The abnormalities become apparent during infancy and affect boys and girls equally. Type II does not become apparent until preadolescence (ages 7 to 11 years) and affect girls more. The abnormality usually does not improve and may develop to severe flexion deformity. Type III is the most severe type involving multiple digits of both upper extremities, and is usually associated with a variety of syndromes [3]. Camptodactyly may also be simplified classified as nonsyndromic camptodactyly (an isolated autosomal dominant anomaly, Type I and Type II) and syndromic camptodactyly (associated with other different manifestations, Type III) [4]. More than 60 distinct types of syndromic camptodactyly have been described in the literature, and camptodactyly phenotype has been observed in carriers with chromosomal abnormalities or gene mutations, including dup(15)(q24q26.3), 22q11 deletion, del(11)(q24.1), microdeletion at 2p15-2p16.1, and the fibroblast growth factor receptor 3 gene (*FGFR3*) p.R621H mutation [4–9]. Only one disease locus (*CAMPD1*) for nonsyndromic camptodactyly with knuckle pads was mapped to chromosome 3q11.2-q13.12, but no disease-causing gene was identified [1]. Here, we report a large Chinese kindred with typical features of isolated fifth finger camptodactyly. The condition is transmitted as an autosomal dominant trait and the talin 2 gene (*TLN2*, MIM 607349) is identified as the disease-causing gene by exome sequencing.

## Materials and Methods

### Pedigree ascertainment and clinical characteristics

**Fig 1:**
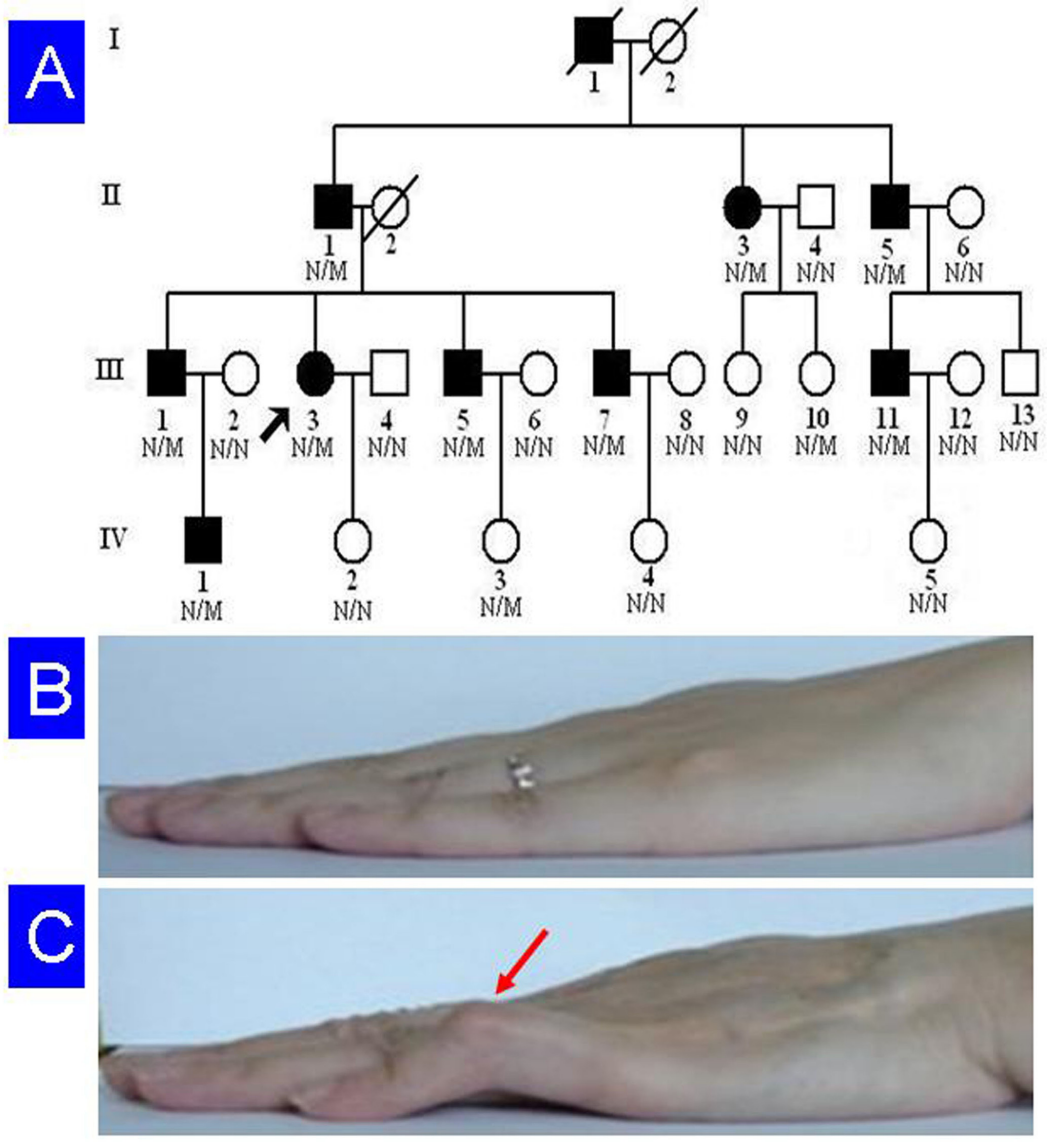
Pedigree of the family with camptodactyly. (A) Pedigree figure. Arrow indicates the proband. N, normal; M, *TLN2* p.S339L mutation. (B) Clinical view of unaffected individual (IV:2). (C) Clinical view of the patient (III:3). Fifth finger contracture is marked with an arrow head.

A four-generation, Chinese Han non-consanguineous pedigree with a total of 26 members, including 10 patients with camptodactyly, was enrolled in this study (Fig 1). Blood was collected from 23 members of this family, including nine patients. They were compared to 1,000 ethnicity-matched normal controls (male/female: 500/500, age 38.9 ± 8.5 years). A detailed medical history and complete physical examination were performed on all family members. Associated defects, such as facial anomalies, contractures of other joints, or skeletal defects were not observed in any family members [1]. The protocol of this study was approved by the Ethics Committee of the Third Xiangya Hospital, Central South University, and each participating individual signed an informed consent. All the methods were carried out in accordance with the principles in the Declaration of Helsinki. According to the pedigree, an autosomal dominant disease-causing gene is likely, since male-to-male transmission was seen.

In this family, camptodactyly presents at birth or in early childhood. The flexion contracture deteriorates slowly over the first two decades of life, and then stabilizes. The abnormality is characterized by bilateral, low-set fifth fingers with contracture at PIP joints. It is typically accompanied by hyperextension at metacarpophalangeal joints, and sometimes at distal interphalangeal joints (Table 1). The degree of flexion varies from slight deformity to almost right-angled bend. None of the affected individuals have knuckle pads on the PIP joints. X-ray of hands does not show any bone or joint abnormality. No foot anomalies are observed in this family. Electrocardiography was performed for all family members and coronary angiography was performed for II:3 (Table 1).

**Table 1:** Camptodactyly phenotype and heart-associated characters in the *TLN2* p.S339L carriers.

Individual	Gender	Age (years)	Left camptodactyly	Right camptodactyly	Knuckle pads	Sinus arrhythmia	Sinus bradycardia	Other cardiac phenotypes
**II:1**	M	76	++	++	–	+	+	–
**II:3**	F	67	–	+	–	+	–	ST segment depression, CMB
**II:5**	M	57	+	+	–	++	–	–
**III:1**	M	57	++	++	–	+	–	–
**III:3**	F	55	+	+	–	–	–	Lowering of T-wave
**III:5**	M	53	+	++	–	++	–	–
**III:7**	M	45	+	+	–	+	–	–
**III:10**	F	42	–	–	–	–	–	–
**III:11**	M	39	–	+	–	+	–	–
**IV:1**	M	30	+	+	–	–	+	–
**IV:3**	F	27	–	–	–	–	+	–

CMB: coronary myocardial bridge; F: female; M: male; ++: prominent in camptodactyly or frequently in heart disease; +: mild in camptodactyly or occasionally in heart disease;–: absent.

### Genotyping and linkage analysis

Genomic DNA was extracted from peripheral blood using standard phenol-chloroform method. Genomewide screening was performed using Linkage Mapping Set Version 2.5 (Applied Biosystems Inc., USA). PCR amplification was performed as previously described [10], and PCR products were appropriately pooled, loaded onto POP4 gel and run in an Applied Biosystems 3500 genetic analyzer. Statistical analysis was performed based on an autosomal dominant disease with a penetrance of 0.90. The frequency of the abnormal allele was set at 1/10,000, in view of the rarity of the disorder. The recombination frequency was assumed to be equal for both sexes. Two-point LOD scores were calculated using Linkage program version 5.1 [10].

### Targeted capture and exome sequencing

Three micrograms of genomic DNA was used to extract the exome library. Genomic DNA from four patients (II:3, III:1, III:3 and III:11, Fig 1) was sheared by sonication and then hybridized to the SureSelect Biotinylated RNA Library (BAITS) for enrichment, according to the manufacturer’s instructions. The enriched library targeting the exome was sequenced on the HiSeq 2000 platform to get paired-end reads with read length of 90 bp [11]. A mean exome coverage of 88.83-fold were obtained and such deep coverage provided sufficient depth to accurately call variants at 99.13% of each targeted exome.

### Read mapping and variant analysis

The human reference genome and the gene annotation were obtained from the UCSC database (http://genome.ucsc.edu/), version hg19 (build 37.1). Alignment of the sequences from the four patients was performed using SOAPaligner after the duplicated reads were deleted, and single nucleotide polymorphisms (SNPs) were called using SOAPsnp set with the default parameters. Insertions and deletions (Indels) affecting coding sequence or splicing sites were identified. The thresholds for calling SNPs and short indels included the number of unique mapped reads supporting a SNP had to be ≥4, and the consensus quality score had to be ≥20 (The quality score is a Phred score, generated by the program SOAPsnp 1.03, quality score 20 represents 99% accuracy of a base call). Common changes of the four patients were obtained by further comparison of the variants of all four cases. All changes were filtered against the SNP database (dbSNP132, http://www.ncbi.nlm.nih.gov/snp/), 1000 genomes data (1000genomes release_20100804), and HapMap (2010–08_phase I + II + III) project, respectively. PCR products were purified as described previously and Sanger sequencing was employed to validate the identified potential disease-causing variants with Applied Biosystems 3500 genetic analyzer [12].

Multiple sequence alignments were performed using the Basic Local Alignment Search Tool (http://blast.st-va.ncbi.nlm.nih.gov/Blast.cgi/). The Sorting Intolerant from Tolerant (SIFT) prediction (http://sift.jcvi.org/, output values less than 0.05 are deleterious), was performed to evaluate whether an amino acid substitution affects protein function [13,14]. Polymorphism Phenotyping version 2 (PolyPhen-2, http://genetics.bwh.harvard.edu/pph2/) and MutationTaster (http://www.mutationtaster.org/) were used to evaluate the possible effects of amino acid substitution on protein structure and function in terms of sequence conservation, chemical change, and likelihood of pathogenicity [15,16]. A three dimension model of the protein was created by PyMOL 1.7 based on the CPHmodels-3.2 [17].

### Cell culture, plasmid transfection and expression analysis

Human embryonic kidney 293 (HEK293) cells (ATCC Number CRL-1573, American Type Culture Collection, USA) were maintained in Dulbecco’s modified eagle medium (DMEM), supplemented with 10% fetal calf serum, penicillin (100 U/ml), streptomycin (100 μg/ml) and amphotericin B (0.25 μg/mL). Cells from passages 10 to 20 were used and they were seeded at 40% confluency into 96-well dish.

Human wild type (WT) *TLN2* cDNA (NM_015059) pCMV6-AC-GFP plasmid was purchased from Origene. *TLN2* p.S339L mutant plasmid was constructed by YRBIO Co. Ltd. (Changsha, China) and was verified by sequencing. Transient transfection of HEK293 cells was performed using 5–10 μg of plasmid DNA and Lipofectamine 2000 (Life Technologies, USA) and following the manufacturer’s instructions. Cells were analyzed 48 h after transfection. Observation and photography of the signals were done with a multifunction microscope (LSM510, Zeiss).

## Results

**Fig 2:**
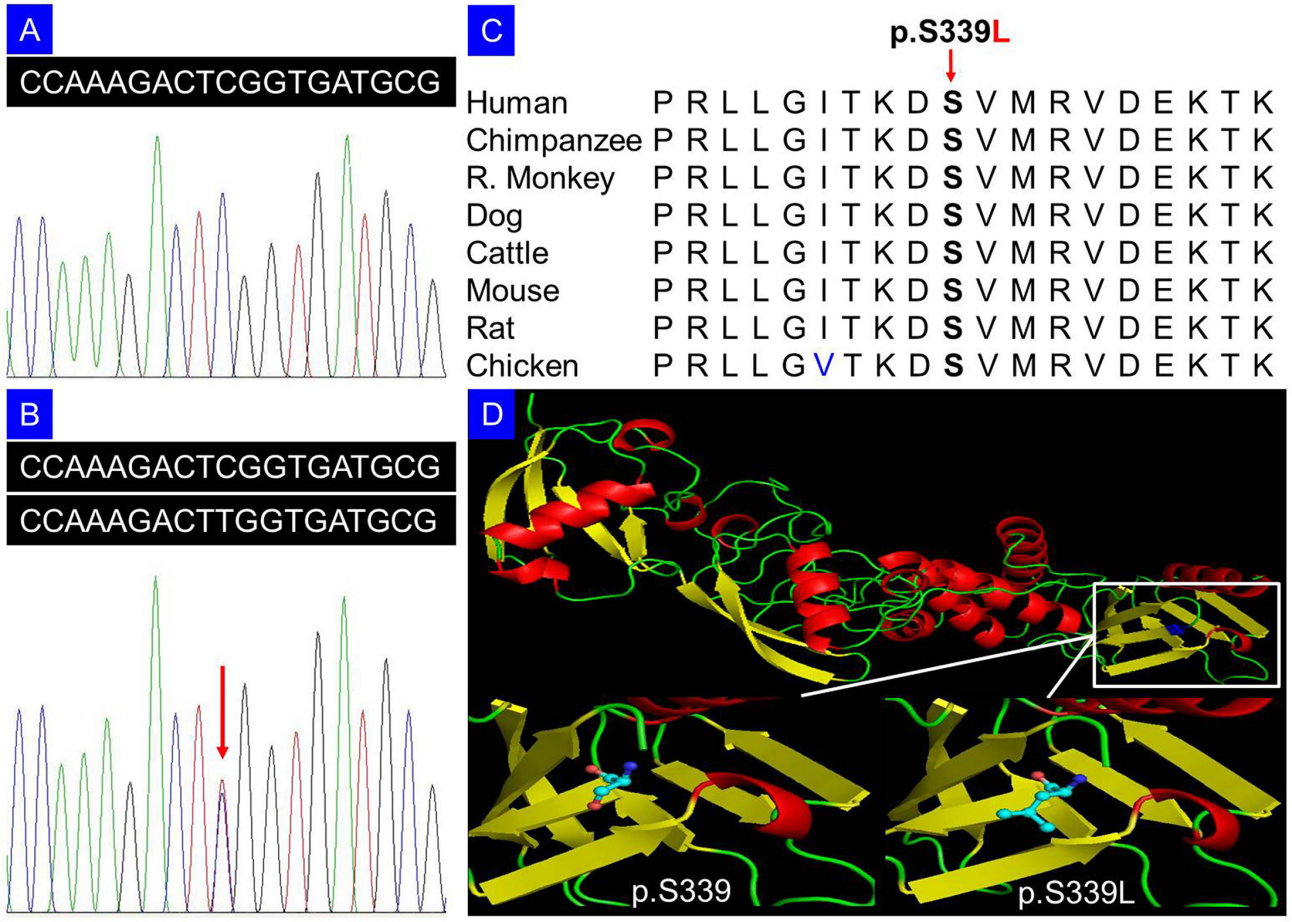
Sequencing analysis of p.S339L mutation in the *TLN2* gene (DNA). (A) Unaffected member (IV:2) of the family. (B) Patient (IV:1) with heterozygous p.S339L mutation. (C) Serine at position 339 is highly conserved across different animal species. (D) Cartoon representation of the model structure of the TLN2 protein by PyMOL 1.7 based on the CPHmodels-3.2: The serine and the mutated leucine at position 339 are shown as ball-and-stick models.

Genome scan supported a disease locus for camptodactyly 2 at chromosome 15q with a maximum LOD score of 2.20 at *D15S131* (*θ* = 0). After excluding known variants identified in dbSNP132, 1000 genomes project, and HapMap with MAF >0.50%, there were 245 variants that were common among these four patients by exome sequencing. We then performed segregation analysis of the available 19 members of the family by Sanger sequencing (Fig 1). Only one variant co-segregated with the disease phenotype in this family: a C to T change in exon 9 (c.1016C>T), resulting in a p.S339L amino acid change in the *TLN2* gene (NCBI Reference Sequence: NM_015059.2, NP_055874.2). The variant was identified in all nine patients and two females without disease phenotype, and was absent in other unaffected family members (Fig 2A and 2B), in 1,000 ethnicity-matched unrelated controls, and in 2,375 ethnicity-matched controls from exome sequencing data from BGI-Shenzhen, indicating it may be a pathogenic mutation. Two asymptomatic individuals carrying this mutation suggested incomplete penetrance which is consistent with the previous reports or more complicated compensated mechanism [18]. Additionally, various relatively benign cardiac phenotypes were observed in most of the p.S339L carriers, including sinus arrhythmia and sinus bradycardia, but absent in other unaffected members without p.S339L mutation (Table 1). Coronary angiography showed coronary myocardial bridge (CMB) in a p.S339L carrier (II:3) with ST segment depression and occasional chest pain. Multi-sequence alignment suggests that the serine at position 339 is phylogenetically conserved among various species (Fig 2C). SIFT prediction revealed a score of 0.01, indicating that the substitution is predicted to affect protein function. PolyPhen-2 analysis produced a score of 0.97 on HumVar database (sensitivity: 0.59; specificity: 0.93), which is predicted to be probably damaging. MutationTaster predicted that the alteration was disease-causing with a probability value close to 1, which indicates the high security of prediction. Cartoon representation of the protein structure was shown (Fig 2D).

**Fig 3:**
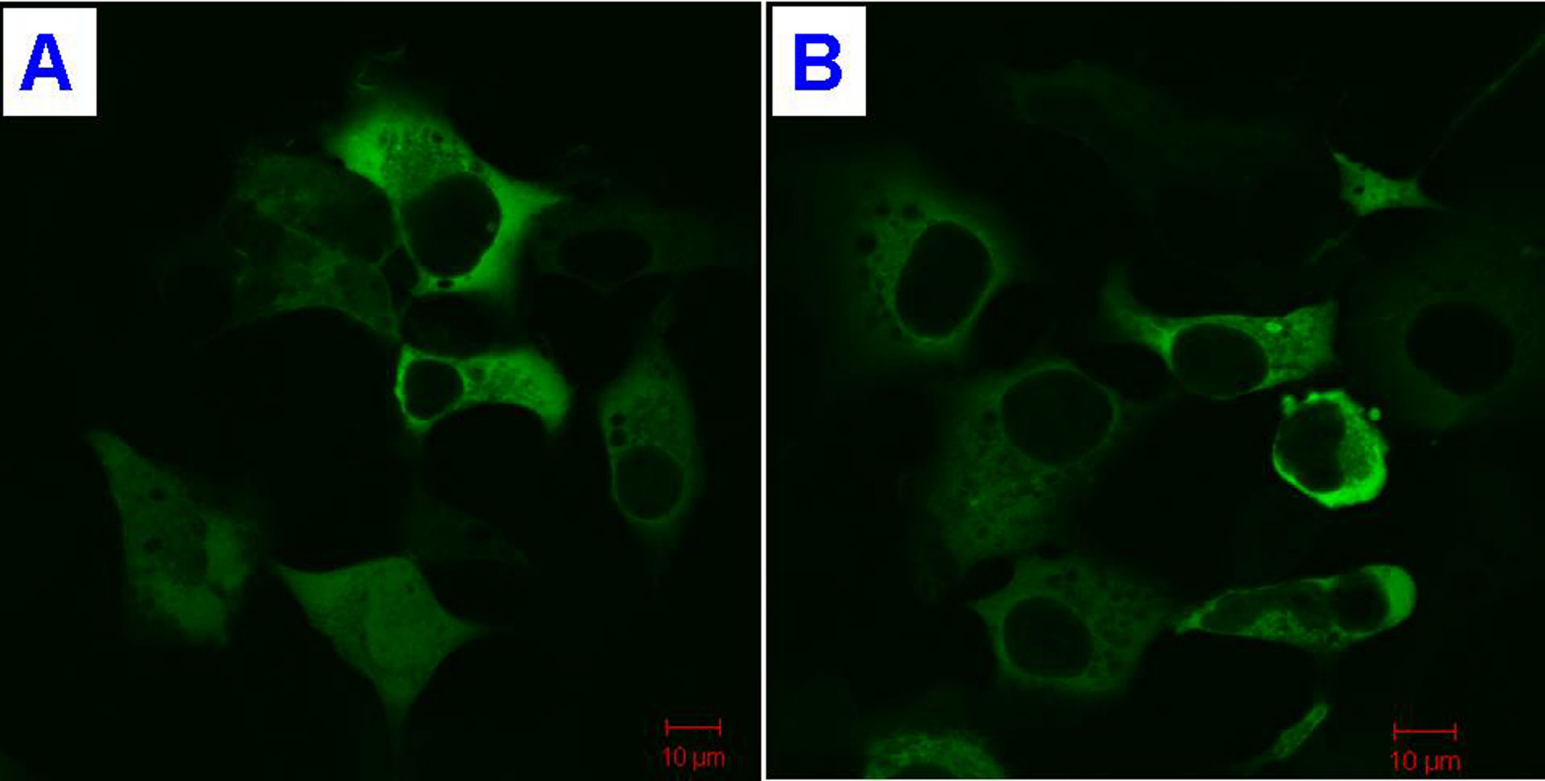
Subcellular localization of TLN2. (A) Wild type TLN2. (B) The p.S339L mutant TLN2.

Protein expression of TLN2 WT and p.S339L mutant was observed by fluorescence microscopy analysis. Expression of wild and mutant TLN2 in HEK293 cells suggests that the protein is predominately cytoplasmic (Fig 3).

## Discussion

Camptodactyly, a non-traumatic, painless and non-neurogenic flexion abnormality at the PIP joint of the fifth finger, may occur in isolation or in various developmental dysmorphology syndromes [19]. Several anatomical abnormalities, including anomalous lumbrical muscle, short flexor digitorum superficialis, retractile subcutaneous tissue and anomalous extensor muscle have been prosecuted as the cause of camptodactyly [20]. It accounts for 5% of congenital hand deformity and 1% of general congenital anomalies [18,21]. Camptodactyly has been postulated to be caused by a dynamic imbalance due to abnormal intrinsic anatomy, and the most common cause is abnormal lumbrical insertion, followed by the shortening of the flexor digitorum sublimis or the central slip of the extensor tendon [22]. Two-thirds of patients have bilateral camptodactyly, although the degree of flexion may not be symmetrical. Most of patients do not seek treatment [3], and standard treatments include complicated open surgery with release, re-insertion/transfer of tendons, and different types of wedge osteotomies [22]. Environmental factors, including infection, trauma and burn may be responsible for a phenocopy. Nonsyndromic camptodactyly may appear to be sporadic in a family, but detailed clinical examination of relatives usually reveals that there may be an autosomal dominant pattern of inheritance, with incomplete penetrance and variable phenotypic expression [1].

In this study, a heterozygous mutation, p.S339L, in the *TLN2* gene was identified in a large Chinese Han pedigree with nonsyndromic camptodactyly. The fifth finger camptodactyly and equal severity between male and female patients suggest that the family has Type I camptodactyly. No obvious knuckle pads observed in this pedigree supported the clinical and genetic heterogeneity, which is not consistent with description in previous reports [1,23,24].

The *TLN2* gene, mapped to chromosome 15q15-q21, contains 66 exons and spans about 190 kb. The deduced 2,557-amino acid protein contains a FERM (four-point-one, ezrin, radixin, moesin) domain. TLN2 consists of an amino-terminal “head” domain linked to a carboxy-terminal “rod” region forming as antiparallel dimmers and it produces multiple transcripts with the highest levels in heart [25]. The FERM domain within the talin head forms the primary molecular link to the β-integrin cytoplasmic tail, though the rod domain may also have some contribution [26].

The cytoskeletal protein, talin, acts as an essential link between integrins and the actin cytoskeleton in several functionally distinct adhesion complexes, including focal adhesions, costameres and intercalated disks [27]. Vertebrates contain two talin proteins (TLN1 and TLN2), and the activity of these proteins increases during muscle regeneration [27,28]. TLN1 is essential for maintaining the connection between integrins and myofilaments at myotendinous junctions, while TLN2 may compensate for some functions of TLN1 [29]. TLN2 protein showed 74% identity and 86% similarity to TLN1 [25], and the difference between TLN1 and TLN2 may confer specific functions, such as different affinity to F-actin [29]. It is induced during striated muscle differentiation and is targeted to stable adhesion complexes in mature muscle [27]. Tln2 diminished in masseter and temporal muscle of mdx mice, a model of Duchenne muscular dystrophy [28]. In cells lacking talin 2, there is no activation of β1 integrin, and loss of the extracellular matrix-integrin-cytoskeleton linkage and sustained cell spreading and adhesion [30].

Genetic heterogeneity is known for nonsyndromic camptodactyly. Nonsyndromic camptodactyly with knuckle pads was mapped to chromosome 3q11.2-q13.12 (*CAMPD1*) [1]. We propose to designate the 15q22.2 locus as *CAMPD2* (i.e., camptodactyly 2), and the *TLN2* gene is prosecuted as the disease-causing gene. Associated clinical features for CAMPD2 include absence of knuckle pads and equal severity between male and female patients.

In this family, the mutation co-segregates with camptodactyly phenotype and two asymptomatic carriers. The latter may be due to incomplete penetrance or more complicated compensated mechanism. Mild relatively benign cardiac conditions including sinus arrhythmia and sinus bradycardia, were observed in family members who carry the p.S339L mutation. This finding is in accordance with the highest expression of TLN2 in heart [25].

In summary, our results not only reveal that *TLN2* mutation is associated with isolated camptodactyly in a Chinese Han family but also confirm genetic heterogeneity in this disorder. Further studies, including identification of genetic and epigenetic factors that modify the *TLN2* gene expression, and generation of appropriate animal models, may help to unravel molecular pathogenesis of this disorder.
